# An Update to Dialysis-Based Drug Release Testing—Data Analysis and Validation Using the Pharma Test Dispersion Releaser

**DOI:** 10.3390/pharmaceutics13122007

**Published:** 2021-11-25

**Authors:** Marc-Phillip Mast, Harshvardhan Modh, Julian Knoll, Elena Fecioru, Matthias G. Wacker

**Affiliations:** 1 Fraunhofer Institute for Translational Medicine and Pharmacology ITMP, Theodor-Stern-Kai 7, 60596 Frankfurt am Main, Germany; mast@em.uni-frankfurt.de; 2 Goethe University, Max-von-Laue-Straße 9, 60438 Frankfurt am Main, Germany; J-Knoll@outlook.com; 3 Department of Pharmacy, Faculty of Science, National University of Singapore, 4 Science Drive 2, Singapore 117544, Singapore; phahbm@nus.edu.sg; 4 Eurofins PHAST Development GmbH & Co. KG, Byk-Gulden-Straße 2, 78467 Konstanz, Germany; elena.fecioru@phast.com

**Keywords:** nanomedicine, release testing, dialysis, semisolids, validation, liposomes, semisolids, creams, topical formulations, dissolution, drug release

## Abstract

Currently, a wide variety of complex non-oral dosage forms are entering the global healthcare market. Although many assays have been described in recent research, harmonized procedures and standards for testing their in vitro performance remain widely unexplored. Among others, dialysis-based techniques such as the Pharma Test Dispersion Releaser are developed for testing the release of drugs from nanoparticles, liposomes, or extracellular vesicle preparations. Here, we provide advanced strategies and practical advice for the development and validation of dialysis-based techniques, including documentation, analysis, and interpretation of the raw data. For this purpose, key parameters of the release assay, including the hydrodynamics in the device at different stirring rates, the selectivity for particles and molecules, as well as the effect of excipients on drug permeation were investigated. At the highest stirring rate, a more than twofold increase in the membrane permeation rate (from 0.99 × 10^−3^ to 2.17 × 10^−3^ cm^2^/h) was observed. Additionally, we designed a novel computer model to identify important quality parameters of the dialysis experiment and to calculate error-corrected release profiles. Two hydrophilic creams of diclofenac, Voltaren^®^ Emulgel, and Olfen^®^ gel, were tested and provide first-hand evidence of the robustness of the assay in the presence of semisolid dosage forms.

## 1. Introduction

For the past decades, drug products comprising fine dispersions of particles have gained a significant market share globally [1,2,3]. Microparticle and nanoparticle drug products are being used by the pharmaceutical industry and changed the requirements in performance testing. Among others, nanomaterial-based formulations are applied in peroral [4,5], parenteral [3], and topical drug delivery [6,7,8]. Recent examples include delivery systems of proteins or peptides, the Sars-Cov-2 mRNA vaccines, as well as extracellular vesicle (EV) preparations [3]. Additionally, a considerable number of conventional dosage forms such as ointments, creams, or gels challenge the compendial drug release assays. These preparations are tested using the Vertical Diffusion Cell, the Immersion Cell, or the Flow-Through Cell described by the United States Pharmacopeia (USP). All these methods are characterized by the formation of a static layer of the dosage form perfused by a limited amount of release medium. Although some of these methods reflect the physiology of the administration route to a certain extent, the instruments provide the operator with very limited control over the release process. This makes it more difficult to apply these compendial release assays in quality control or drug development.

**Figure 1:**
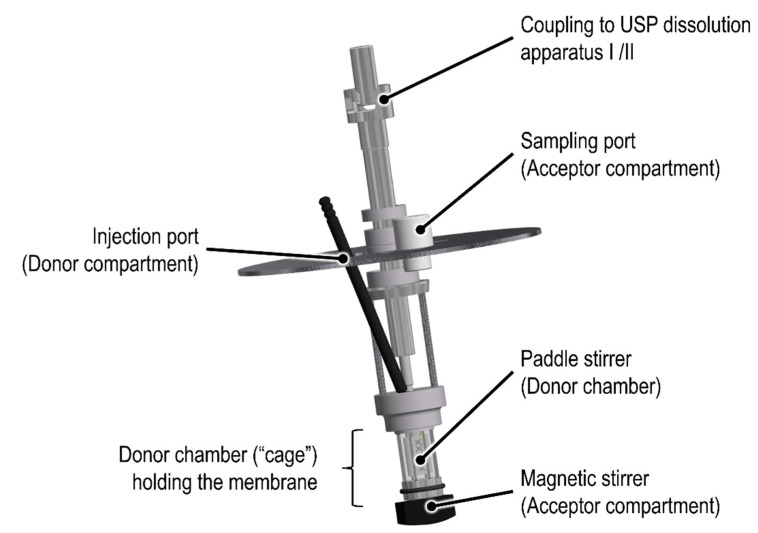
Schematic of the commercial Pharma Test Dispersion Releaser (PTDR).

To date, there are several dialysis-based quality control methods established that enable a highly selective separation of fine particles from the release medium [9]. Among others, the Float-A-Lyzer^®^ (Repligen, Waltham, MA, USA) in combination with USP dissolution apparatus I/II, the USP apparatus IV Dialysis Adapter (Sotax AG, Aesch, Switzerland), and the Pharma Test Dispersion Releaser (PTDR) (Pharma Test Apparatebau AG, Hainburg, Germany) have been described. Initially developed by Wacker and Janas at Goethe University [10], the commercial PTDR (Figure 1) is manufactured by Pharma Test Apparatebau AG (Hainburg, Germany). It is used in combination with a USP dissolution apparatus I/II. While the donor compartment is formed by a small cage holding the dialysis membrane (Figure 1), the dissolution vessel represents the acceptor compartment. The donor and acceptor compartments are constantly agitated at a similar rate.

The instrument was successfully used to investigate the drug release from liposomes [11,12,13], nanoparticles [5,14,15], microcrystals [16], microspheres [17], and extracellular vesicle (EV) preparations. For many applications, the capability of the assay to predict the in vivo performance was confirmed by correlation of the in vitro and in vivo release rate [3,5,11,12,14,16,17]. In the present work, we emphasize differences in the workflow with regards to parameter selection (e.g., stirring rate, membrane material), reference experiments, data evaluation, and data interpretation, when dialysis-based in vitro release tests are developed [9,18,19].

**Figure 2:**
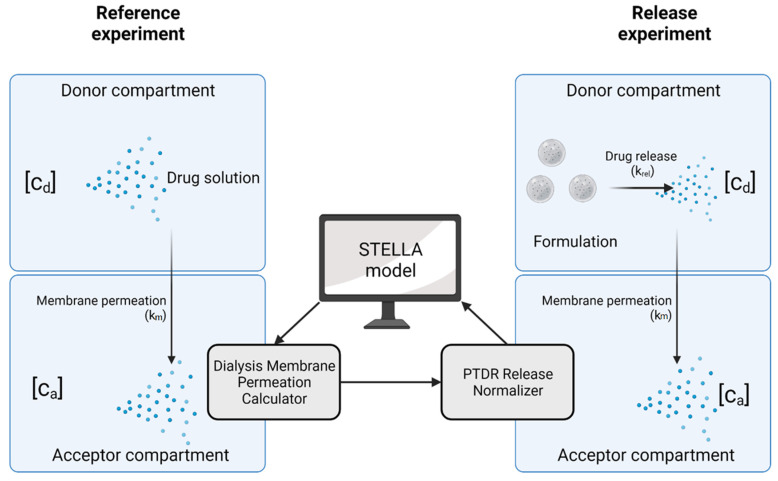
Kinetic processes involved in dialysis. The membrane permeation rate (*k_m_*) is determined in a reference experiment using a drug solution. This membrane permeation rate is later applied in the normalization of the release profile. In this illustration [c_d_] represents the drug concentration in the donor compartment and [c_a_] the drug concentration in the acceptor compartment. Created with BioRender.com.

Dialysis involves two kinetic processes with an impact on the release profile, the release kinetics of the drug substance from the carrier (described by the release rate constant *k_rel_*), and the permeation rate through the dialysis membrane (described by the membrane permeation rate constant *k_m_*). Both processes, together with an illustration of their quantification, are presented in Figure 2. In the following, we lay out a recommended practice and provide an overview of the technical operations that confirm the functionality of the PTDR under various conditions. In addition, we outline reference experiments that minimize the expected analytical errors.

To harmonize the treatment of dialysis-based release data, we used the visual programming language Systems Thinking, Experimental Learning Laboratory with Animation (STELLA) to provide a user-friendly interface for the calculation of the membrane permeation rate constant (*k_m_*) and normalization of the release profiles. The dialysis membrane permeation calculator (DIMEC) and the PTDR Release Normalizer (ReNo) are available under the Creative Commons License. 

## 2. Materials and Methods

### 2.1. Chemicals and Materials

Spectra/Por^®^ 6 regenerated cellulose (RC) dialysis membranes with a molecular weight cut-off (MWCO) of 50 kDa and a flat diameter of 28 mm were purchased from VWR International GmbH (Darmstadt, Germany). A stabilized suspension of reactant-free gold nanoparticles with a diameter of 50 nm (Sigma-Aldrich Chemie GmbH, Munich, Germany) in 0.1 mM phosphate-buffered saline (PBS) was used for the membrane leakage test. Diclofenac diethylamine (DEA) salt (Cayman Chemical, Ann Arbor, MI, USA) was purchased from Biomol GmbH (Hamburg, Germany) and diclofenac sodium salt was obtained from Sigma-Aldrich Chemie GmbH (Munich, Germany). Olfen^®^ gel (10 mg/g diclofenac sodium, Mepha Pharma AG, Basel, Switzerland), and Voltaren^®^ Emulgel (11.6 mg/g diclofenac-DEA, GlaxoSmithKline Consumer Healthcare GmbH & Co. KG, Munich, Germany) were obtained from a retail pharmacy. Idebenone was purchased from Rxn Chemicals (Hadapsar, India). All the other chemicals were of analytical grade or equivalent and used as received. An Ultra Clear^®^ system (Evoqua water technologies, Günzburg, Germany) was used for water purification in all the experiments.

### 2.2. High-Performance Liquid Chromatography 

The diclofenac concentrations were quantified using a Chromaster high-performance liquid chromatography (HPLC) system (VWR Hitachi, Tokyo, Japan). An HPLC pump (5160), a column oven (5310), an autosampler (5260), and a UV-Vis detector (5420) were used. The mobile phase consisted of 62% (*v*/*v*) of acetonitrile and 38% (*v*/*v*) of 0.1% (*v*/*v*) of trifluoroacetic acid in ultrapure water. Separation was carried out in a reversed-phase column (Gemini NX-C18, 5 µm, 250 × 4.60 mm, 110 A, Phenomenex Ltd., Aschaffenburg, Germany). The flow rate was set to 1 mL/min and a temperature of 30 °C was maintained over the run time of 12 min. The detection wavelength was 276 nm [20].

The samples containing bovine serum albumin (BSA) were diluted with the sample solvent comprising 62% (*v*/*v*) of acetonitrile and 38% (*v*/*v*) of 0.01% (*v*/*v*) of trifluoroacetic acid in ultrapure water. They were incubated for 30 min (750 rpm, 16 °C) using a Thermal Shake lite (VWR International GmbH, Darmstadt, Germany). The reduced amount of acid was used to prevent the degradation of the diclofenac. Next, the precipitated protein was removed by centrifugation (14,000× *g* rpm, 15 min, 16 °C) using a Centrifuge 5430× *g* R with an FA-45-30-11 rotor (Eppendorf AG, Hamburg, Germany). The supernatant was transferred into HPLC vials and a volume of 40 µL was injected into the HPLC system. The samples without proteins were diluted similarly and a volume of 40 µL was injected without further treatment. The linearity was demonstrated in a concentration range from 0.12–30 µg/mL for both the diclofenac salts. The limit of detection (LOD) and limit of quantitation (LOQ) were determined to be 24 and 80 ng/mL, respectively.

### 2.3. Size Exclusion Chromatography

The BSA was quantified using a Chromaster HPLC system (VWR Hitachi, Tokyo, Japan) equipped with a pump (5160), an autosampler (5260), a column oven (5310), and a UV-Vis detector module (5420). The flow rate was set to 0.9 mL/min and a detection wavelength of 280 nm was used. The column temperature was adjusted to 25 °C. A PBS solution (pH 6.8, 0.25% sodium azide) was used as the mobile phase. A volume of 50 µL was injected into the HPLC system. For this separation, a Yarra SEC-3000 column (3 µm, 300 × 7.8 mm, 200 A, Phenomenex Ltd., Aschaffenburg, Germany) was used. Linearity was demonstrated in a range from 20–500 µg/mL.

### 2.4. Drug Solubility

The solubilities of diclofenac-DEA and diclofenac sodium were determined in different media with a modified shake-flask method. A volume of 4 mL of each medium was filled into glass vials with an excess of the drug substance. The vials were incubated at 37 °C (INCU-Line^®^ Standard IL 68 R, VWR International GmbH, Darmstadt, Germany) for 24 h under constant stirring with a multi-position magnetic stirrer (Cimarec™ Poly 15, Fisher Scientific GmbH, Schwerte, Germany) at 550 rpm. After 24 h, the solid excess was separated with a polytetrafluoroethylene (PTFE) syringe filter (13 mm diameter, 0.45 µm pore size, VWR International GmbH, Darmstadt, Germany). The filtrates were diluted and analyzed by HPLC (see Section 2.2). The PTFE filters were saturated with 1 mL of drug suspension before the samples were collected. These solubility experiments were carried out at 32 °C and all the experiments were performed in triplicates.

### 2.5. Technical Operations

All the experiments were carried out with a commercial PTDR system (Pharma Test Apparatebau AG, Hainburg, Germany) in a PTWS 120 S dissolution apparatus II (Pharma Test Apparatebau AG, Hainburg, Germany) following the specifications of the USP. A mini-vessel (250 mL) following the specifications of the Chinese Pharmacopeia [21] was used as the acceptor compartment. The vessels were filled with 120 mL of filtered and degassed (40 °C, 300 mbar, 30 min) medium. The membrane was pre-treated according to the instructions of the manufacturer, mounted around the donor chamber, and sealed with two O-rings. The volume added to the donor compartment was 3.4 mL, leading to a total volume of 123.4 mL in the final setup. The sampling volume, time points, temperature, and stirring rates are described in the later sections of this article. The donor chamber featured a surface area of 10.95 cm^2^. The thickness of the RC membrane was 0.0065 cm.

#### 2.5.1. Membrane Leakage Test Using Gold Nanoparticles

The PTDR setup is commonly used for testing the drug release from fine particles or vesicles. The membrane leakage test evaluates the retention of the particle population of interest by the membrane. Gold nanoparticles with a diameter of 50 nm were used as a standard. A volume of 3.4 mL of the liquid dispersion was injected into the donor chamber which was sealed by an RC membrane (50 kDa). PBS (0.1 mM) was filled into each mini-vessel and samples with a volume of 1 mL were collected after 1, 2, 4, 8, and 24 h. After 24 h, the RC membrane was punctured with a scalpel blade and, after 30 min, a 1 mL sample was collected. Additionally, one negative control (a setup without the addition of gold nanoparticles into the donor chamber) and one positive control (a setup with the membrane being punctured at the beginning of the experiment) were tested. The samples were analyzed using a Zetasizer Nano ZS (Malvern Instruments GmbH, Herrenberg, Germany). Additionally, the particle concentration was measured at a wavelength of 535 nm in a UV-Vis spectrophotometer (U-3000 Spectrophotometer, Hitachi, Tokyo, Japan). The dissolution apparatus operated at 37 ± 0.5 °C and 100 rpm. 

Before quantification by UV-Vis spectroscopy, a wavelength scan was performed in a range from 300–700 nm at a rate of 300 nm/min, and linearity was confirmed for the expected concentration range. To use the Zetasizer Nano ZS to determine gold nanoparticles quantitatively, the derived photon count rate was used. For monodispersed nanoparticles smaller than the laser wavelength (633 nm), the intensity of the scattered light I is proportional to the concentration of the nanoparticles in the sample [22,23]. This mathematical relationship is described by the Rayleigh equation (Equation (1)):

(1) $$I=I_{0}\cdot \alpha \cdot \frac{m^{2}-1}{m^{2}+2}\cdot d^{6}\cdot c$$

where *I*_0_ is the incident light intensity, *α* is an instrument coefficient, *m* is the refractive index, *d* is the particle diameter, and *c* is the particle concentration. The instrument measures the derived photon count rates, which are a surrogate for the scattered light intensity. It can be used to estimate the particle concentration. The measurements were conducted in triplicate at a fixed position in the center of the cuvette using the parameter summarized in Table 1. Before each measurement, the temperature was equilibrated for 120 s to 25.0 °C. Linearity was confirmed in the expected range of dilution.

These parameters are commonly used by the dynamic light scattering (DLS) system for a concentration-independent calculation of the particle size. However, in the present approach, we used the derived photon count rate to determine particle concentrations. Because of the specifics of these measurements, any reproduction of our findings requires this exact configuration.

#### 2.5.2. Retention of Macromolecules

Dialysis membranes are commonly characterized by their MWCO. This parameter describes the molecular size of a marker molecule retained by the membrane over a defined period. For the RC membrane with an MWCO of 50 kDa, at least 90% of BSA with a molecular weight of 66 kDa is retained over 17 h. Protein retention was therefore used to determine the leakage from the PTDR as well. An amount of 100 mg bovine serum albumin (BSA) in 3.4 mL PBS 7.4 was injected into the donor chamber. The acceptor compartment was filled with 120 mL PBS 7.4. The dissolution tester was operated at 37 °C and 50 rpm. Samples (0.2 mL) were collected after 1, 2, 3, 4, 6, 17, 20, and 24 h followed by replenishing the volume with fresh medium. The albumin was quantified using size exclusion chromatography (SEC, Section 2.3).

Additionally, a real-time permeation profile was recorded under similar conditions. The albumin was quantified using an online dip probe UV-Vis spectroscopic measurement system (TIDAS L 520 UV-NIR, Pharmatest Apparatebau AG, Hainburg, Germany). The absorption was measured in a range from 200–400 nm every 5000 ms (iteration time) and an average absorption was calculated every 25 s for 24 h. Reference measurements were conducted, and a linear range was observed ranging from 25–500 µg/mL at 277 nm.

#### 2.5.3. Hydrodynamics in the Acceptor Compartment

In the PTDR, the donor and acceptor compartments are stirred at similar rates. The acceptor compartment has a larger volume and efficient mixing is required to accurately detect the release. Therefore, the mixing efficiency in the acceptor compartment was evaluated by adding the dye idebenone to the acceptor compartment and measuring the concentration with the online dip probe UV-Vis spectroscopic measurement system (TIDAS L 520 UV-NIR, Pharmatest Apparatebau AG, Hainburg, Germany). For this experiment, a 2% (*m*/*v*) sodium dodecyl sulfate (SDS) solution was used as the medium. A volume of 120 mL was filled into the acceptor chamber while 3 mL were added to the donor compartment. The online dip probe was aligned parallel to the center of the donor chamber and the measurement was started as soon as 37 ± 0.5 °C was maintained. One measurement was performed every 1000 ms (iteration time), averaging every 5 s in one value. A wavelength range of 200–650 nm was used. The total run time was 15 min. After 30 s, 0.6 mL of a 5 mg/mL idebenone solution in 2% sodium dodecyl sulfate was added to the acceptor compartment. The mixing efficiency was evaluated at stirring rates of 0, 25, 50, and 100 rpm. Linearity for the quantification of idebenone at 283 nm was demonstrated between 0.9–25 µg/mL.

#### 2.5.4. Drug Permeation Studies at Different Stirring Rates

In dialysis, drug permeation studies are important reference experiments when testing drug formulations for their release properties. The permeation experiments were carried out with diclofenac sodium in PBS at 37 °C and stirring rates of 0, 25, 50, and 100 rpm. The acceptor compartment was filled with 120 mL of PBS. An amount of 5 mg of the drug dissolved in a volume of 3.4 mL of PBS was used and injected into the donor compartment. The chamber was sealed by an RC membrane (50 kDa MWCO) and two O-rings. The PTDR was mounted into the USP dissolution apparatus II (Pharma Test Apparatebau AG, Hainburg, Germany). The experiments were conducted at 37 ± 0.5 °C. Samples (0.2 mL) were collected after 0.125, 0.25, 0.5, 1, 2, 3, 4, 5, 6, 7, and 8 h and the volume was replenished with fresh medium. At the end of each experiment, the samples were collected from the donor compartment to ensure that the equilibrium between the donor and acceptor compartments had been reached. The experiments were repeated with 10 mM phosphate buffer pH 7.4 at 32 °C and 50 rpm for normalization (see Section 3.3.5).

Later steps involved modeling the drug distribution between the donor and the acceptor compartment. To validate our model, we determined the drug concentration after 0.25, 0.5, and 2 h at 100 rpm. The samples were analyzed as described in Section 2.2.

#### 2.5.5. Selectivity of the Assay for Specific Size Fractions

The selectivity of the PTDR setup for different molecular sizes enables the retention of molecules bound to proteins in the donor chamber. In a previous investigation, we highlighted the application of dialysis-based separation to distinguish between drug release and the direct transfer of drug molecules from colloids to proteins [13]. To evaluate the separation on a molecular level, the fraction of diclofenac retained by two different concentrations of BSA was tested. The acceptor compartment was filled with 120 mL of PBS comprising 1 and 10 g/L of BSA, respectively.

An amount of 5 mg of the drug dissolved in a volume of 3.4 mL of PBS was injected into the donor compartment. The chamber was sealed by an RC membrane (50 kDa MWCO) and two O-rings. The experiments were conducted at 37 ± 0.5 °C and 100 rpm. Samples (0.2 mL) were collected after 0.125, 0.25, 0.5, 1, 2, 3, 4, 5, 6, 7, and 8 h and the volume was replenished with fresh medium. The samples were analyzed as described in Section 2.2.

### 2.6. Performance Testing

#### 2.6.1. Evaluation of the Influence of Excipients on the Drug Permeation

Semisolid dosage forms are well known for their interactions with surfaces and membranes. Therefore, we evaluated the PTDR method with regards to potential changes in the detected release due to membrane-excipient interactions. For this purpose, we carried out a release experiment using an emulsion gel, followed by the injection of a drug solution into the acceptor compartment.

An amount of 430 mg of Voltaren^®^ Gel, which corresponds to 5 mg diclofenac-DEA, was weighed into the donor chamber and the release medium was added to a total volume of 3.4 mL. The acceptor compartment was filled with 120 mL phosphate buffer pH 7.4 as the release medium. An RC membrane (MWCO 50 kDa) was used and the dissolution apparatus was operated at 32 ± 0.5 °C and 50 rpm. Samples (0.2 mL) were collected after 0.25, 0.5, 1, 2, 3, 4, 5, 6, 7, 8, and 24 h, and the volume was replenished with fresh medium. Afterward, an amount of 5 mg diclofenac-DEA dissolved in 0.5 mL of release medium was added to the donor chamber, and samples were collected as described above. The samples were analyzed as described in Section 2.2.

#### 2.6.2. Comparative Release Studies of Two Semisolid Dosage Forms

Each vessel (acceptor compartment) was filled with a volume of 120 mL medium of a 40 mM acetate buffer pH 5.3. Olfen^®^ and Voltaren^®^ gels were weighed accurately into the donor chamber corresponding to 5 mg of diclofenac salt. Release medium was added to prefill the donor chamber to a total volume of 3.4 mL to avoid diffusion into the donor chamber. The dissolution tester operated at 32 ± 0.5 °C and 50 rpm. Samples (0.2 mL) were taken at 0.25, 0.5, 1, 2, 3, 4, 5, 6, 7, 8, 24, 26, 28, 32, and 48 h and the volume was replenished with fresh medium. The samples were analyzed as described in Section 2.2.

### 2.7. Data Analysis and Computer Model

The evaluation of release data obtained by dialysis experiments often requires a correction of the expected analytical error due to the influences of membrane permeation on the release rate. In the following section, we describe data treatment and evaluation using the four-step model [19]. Two different calculations are made using STELLA. The first calculation identifies the membrane permeation rate constant (*k_m_*) for a given experimental design and drug permeation profile. The operator uses the reference experiment with the dissolved drug substance being added to the donor compartment. A second calculation uses this permeation rate to normalize the release profile. Both STELLA models were published under Creative Commons License.

#### 2.7.1. Modelling Drug Permeation and Normalization

To calculate *k_m_*, the dissolved drug is added to the donor compartment, followed by quantification from the acceptor compartment. The four-step model assumes the diffusion of the drug substance through the membrane to follow Fick’s law of diffusion [15]. It depends on the concentration gradient between the donor and the acceptor compartment (∆*C*), the volume of the acceptor compartment (*V_a_*), the thickness of the dialysis membrane (*δ*), and the surface area of the dialysis membrane separating both compartments (*A*).

(2) $$\frac{dC_{a}}{dt}=\left[\frac{k_{m}\cdot A}{\delta \cdot V_{a}}\right]\times \left[\Delta C\right]$$

For the calculation of the surface area of the dialysis membrane (*A*), the specifications of the PTDR donor cell (radius *r**_d_*** and height *h**_d_*** of the cage cylinder) can be used (Equation (3)).

(3) $$A=2\pi \cdot r_{d}\cdot h_{d}$$

The thickness of the membrane (*δ*) depends on the membrane material and is a common specification reported by the manufacturer. The volume in the acceptor compartment *V_a_* depends on the size of the vessel, used for the drug release test. In the present investigation, a 250 mL mini-vessel configuration following the specifications of the Chinese Pharmacopeia [21] was used, resulting in a total volume of 120 mL in the acceptor compartment. Furthermore, the evaporation of liquid from the dissolution vessel was taken into consideration. Here, we assumed a linear evaporation process over time and corrected *V_a_* for each of the calculated time points. The STELLA model interface uses the initial and the final vessel weight [g] for this calculation. Hence, an identical initial and final vessel weight leads to uncorrected permeation profiles. During the permeation experiment, the concentration of the drug is quantified from the acceptor compartment (*C_a_*). Together with the drug amount injected into the PTDR (*Q*_0_), the concentration in the donor compartment (*C_d_*) can be calculated:

(4) $$C_{d}\left(t\right)=\frac{\left[Q_{0}-C_{a}\left(t\right)\cdot V_{a}\left(t\right)\right]}{V_{d}}$$

Replacing the term *C_d_*(*t*) with Equation (4) in Equation (2) leads to:

(5) $$\frac{dC_{a}}{dt}=\left[\frac{k_{m}\cdot A}{\delta \cdot V_{a}}\right]\cdot \left[\frac{Q_{0}-C_{a}\left(t\right)\cdot V_{a}\left(t\right)}{V_{d}}-C_{a}\left(t\right)\right]$$

Equation (5) can then be solved analytically as follows:

(6) $$C_{a}\left(t\right)=\left[\frac{Q_{0}}{V_{a}\left(t\right)+V_{d}}\right]\cdot \left\{1-\exp\left[\frac{A\cdot k_{m}\cdot \left(V_{a}\left(t\right)+V_{d}\right)\cdot t}{\delta \cdot V_{a}\left(t\right)\cdot V_{d}}\right]\right\}$$

For further calculations, Equation (6) was simplified:

(7) $$C_{a}\left(t\right)=C_{\infty }\cdot \left[1-\exp\left(k_{T}\cdot t\right)\right]$$

with *C*_∞_ being the equilibrium concentration, the equation can now be solved resulting in the newly introduced total diffusion coefficient *k_T_* (Equation (8)).

(8) $$k_{T}=\frac{\ln\left(1-\frac{C_{a}\left(t\right)}{C_{\infty }}\right)}{t}=\left[\frac{A\cdot k_{m}\cdot \left(V_{a}\left(t\right)+V_{d}\right)}{\delta \cdot V_{a}\cdot V_{d}}\right]\;$$

Stella Architect^®^ uses linear extrapolation to create a continuous profile from the data points provided by the operator. The computer model calculates one *k_T_* value every 3 s, including measured and extrapolated time points. The values *k_m_* and *k_T_* remain constant for the same dialysate, and experimental conditions (membrane, stirring rate, temperature, medium) [19]. For each *k_T_*, *k_m_* can be calculated (Equation (9)) as follows:

(9) $$k_{m}=\left[\frac{k_{T}\cdot \delta \cdot V_{a}\cdot V_{d}}{A\cdot \left(V_{a}\left(t\right)+V_{d}\right)}\right]$$

However, deviations from the assumed first-order permeation are more likely to occur during the early time points and in the plateau phase. Therefore, an average membrane permeation rate constant was calculated in a permeation range from 15–85%:

(10) $$k_{m-Average}=\frac{1}{n_{15\%-85\%}}\cdot \overset{15\%}{\underset{85\%}{\;\sum }}k_{m}$$

The second calculation uses the average *k_m_* to calculate the concentration profile in the donor chamber. To calculate the concentration in the donor compartment, the slope is continuously calculated from every two data points [19]:

(11) $$\frac{dC_{a}}{dt}\approx \frac{\Delta C_{a}}{\Delta t}=\left[\frac{k_{m}\cdot A}{\delta \cdot V_{a}}\right]\cdot \left[C_{d}\left(t\right)-C_{a}\left(t\right)\right]$$

The concentration in the donor compartment is calculated for the acquired data points [19]:

(12) $$C_{d}\left(t\right)=\left(\frac{\Delta C_{a}}{\Delta dt}\right)\cdot \left[\frac{\delta \cdot V_{a}}{k_{m}\cdot A}\right]+C_{a}\left(t\right)$$

Finally, the total quantity of released drug from the formulation (*Q_r_*) is determined:

(13) $$Q_{r}\left(t\right)=C_{d}\left(t\right)\cdot V_{d}+C_{a}\left(t\right)\cdot V_{a}$$

#### 2.7.2. Validation of Drug Permeation Model

The membrane permeation rate constant *k_m_* is applied to estimate drug concentrations in the donor and the acceptor compartment over time. To validate our in silico model of the dialysis process, we calculated *k_m_* for the permeation of diclofenac from the drug permeation profile at three different stirring rates (25, 50, and 100 rpm) and compared the predicted with the observed concentration. The absolute average fold error (*AAFE*) was calculated using Equation (14) [11,14,24,25,26,27] with n time points. Additionally, a comparison between the predicted and the observed drug concentration in the donor compartment was made after 0.25, 0.5, and 2 h at a stirring rate of 100 rpm.

(14) $$AAFE=10^{\frac{1}{n}\cdot \sum |\;\log\;\frac{predicted\;value_{t}}{observed\;value_{t}}\;|}$$

## 3. Results and Discussion

Currently, a rising number of complex non-oral dosage forms enter the global healthcare market. As a consequence, there is a need for novel in vitro methodologies to evaluate their quality and safety. Dialysis-based release assays often require significant expertise. Changes in the membrane quality, as well as the influence of the dialysis rate on the release profile, are the most common challenges reported in recent literature.

However, these “urban legends” often ignore other influences on reproducibility, such as the poor standardization of the instrument and release conditions. The PTDR enables in vitro release testing of liquid and semisolid dispersions in a well-defined setup. After normalization of the release profile using the membrane permeation rate constant *k_m_*, these release profiles are widely unaffected by the dialysis rate and enable an improved comparison between different formulations and formulation qualities. Our present investigation provides an overview of the technical operations that confirm the functionality of the PTDR and serves as a guide for experimental design, validation, data evaluation, and documentation of dialysis-based release experiments.

### 3.1. Solubility of Diclofenac Salts in Different Release Media

The equilibrium solubility of diclofenac in various media has an impact on permeation and release experiments. When determining the membrane permeation rate constant *k_m_*, the permeation of a drug solution is measured. Hence, the drug substance must be dissolved completely, and sink conditions must be maintained in the donor chamber as well as in the total volume used for the release assay. Table 2 summarizes the solubilities determined in various release media.

To achieve high aqueous solubility, most of the permeation experiments were carried out in PBS at a pH of 7.4 in the presence or absence of BSA. After the addition of 1 g/L of BSA, there was no significant increase in the solubility of diclofenac sodium observed (ANOVA) as compared to PBS alone. This seems unsurprising considering the low BSA concentration leading to the complexation of approximately 0.7% of the drug. Only at the higher BSA concentration, a significant increase was observed (ANOVA, *p* < 0.05). The release of diclofenac from the two semisolid dosage forms was carried out at lowered temperature (32 °C). Solubilities of diclofenac sodium and diclofenac-DEA in 10 mM phosphate buffer differed significantly (ANOVA, *p* < 0.05), while the difference in 40 mM acetate buffer was negligible. However, the lowered solubility at acidic pH made the detection of differences more challenging. In summary, the expected influences of pH, temperature, and salt form on drug solubility were found.

### 3.2. Technical Performance of the PTDR

Before the release of drug formulations was tested, we carried out a number of technical operations with the PTDR. These included a membrane integrity test, the retention of macromolecules, an evaluation of the hydrodynamics in the acceptor compartment as well as a measurement of the selectivity of the method for the size fraction of interest.

#### 3.2.1. Membrane Leakage Test Using Gold Nanoparticles

The membrane integrity test requires a stable particle standard that can be reliably quantified from the acceptor compartment. Serving as an indicator for the particle populations retained by the dialysis membrane, a monodisperse size distribution was one key criterium for the selection. Among other potential standards, gold nanoparticles led to the most reliable outcome. When developing this protocol, a number of commercial nanobeads and polymer nanoparticles were tested (Supplementary Table S1). The limited stability of these preparations was one of the most common weaknesses and included agglomeration or release of the dye.

**Figure 3:**
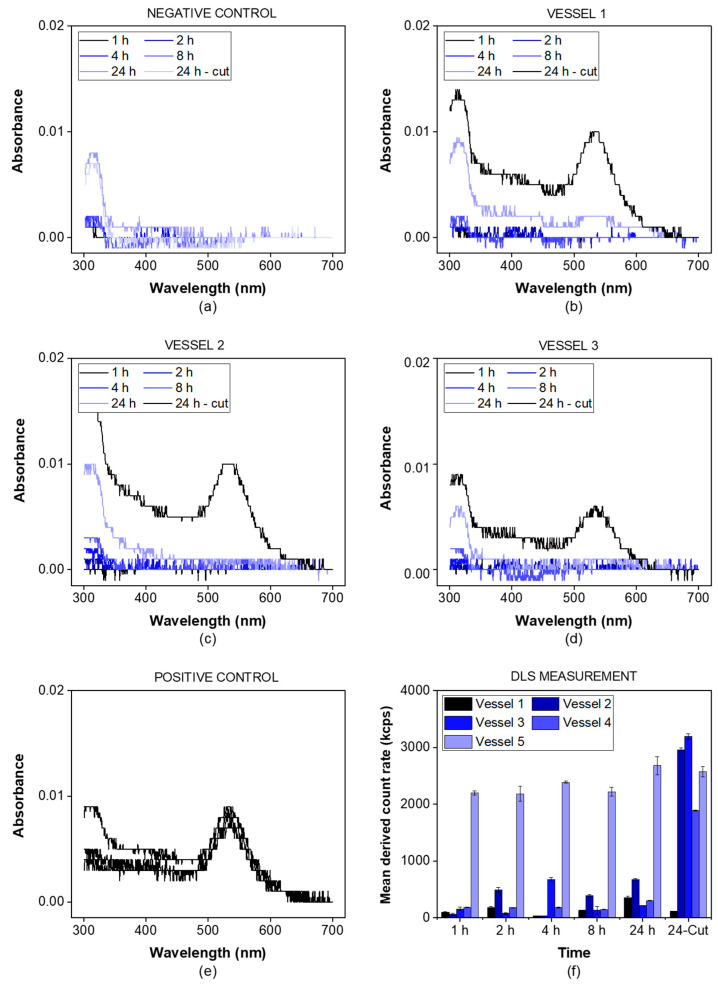
Membrane integrity test using a dispersion of gold nanoparticles (λ_max_ = 535 nm). The samples were analyzed by UV-VIS spectrophotometry at a wavelength of 300–700 nm. The negative control was measured without adding gold nanoparticles (**a**) while the positive control involved an incision through the membrane before starting the experiment (**e**). For all other vessels (**b**–**d**), the gold nanoparticles were added to the vessel and the spectrum was recorded at the indicated time points. After 24 h, a small incision through the membrane was performed. The spectra indicating the presence of gold nanoparticles in the acceptor compartment were highlighted in black for all vessels. Additionally, the mean derived count rate in the acceptor compartment was measured indicating the presence of gold nanoparticles in all vessels after the incision but not before this time point, except for the positive control (**f**).

The gold nanoparticles exhibited a particle diameter of 50 nm, representing a suitable lower size limit for many drug delivery systems. The particles were detected photometrically and by using the derived photon count rate in a DLS setup. The negative control was measured in the absence of gold nanoparticles (Figure 3a), while for the positive control, a small incision in the dialysis membrane allowed the particles to leak into the acceptor chamber (Figure 3e).

For all three vessels (Figure 3b–d), the absorbance of gold nanoparticles was detected after the incision only (24 h sample). This indicates that there was no leakage from the donor into the acceptor compartment over the whole duration of the experiment. The nanoparticles were successfully retained by the dialysis membrane. This was also confirmed by the mean derived photon count rate, detected by the DLS setup (Figure 3f). For the given particle size and laser wavelength, the derived count rate served as a surrogate parameter for the particle concentration. It was below 1000 kcps for all vessels before the incision and increased to values of more than 2000 kcps after the incision, except for the positive control. This provides strong evidence that particles with a size of 50 nm are retained by the membrane and do not leak into the acceptor compartment.

#### 3.2.2. Retention of Macromolecules

**Figure 4:**
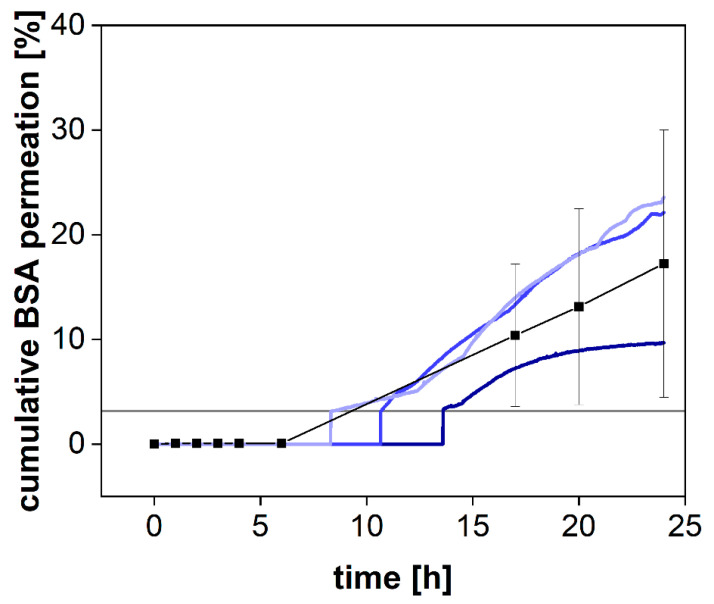
Cumulative permeation of bovine serum albumin (BSA) using SEC-HPLC method (black squares, Mean ± SD, *n* = 3) or an online UV-VIS dip probe (blue colored lines) at 37 °C and 50 rpm using a 50 kDa RC dialysis membrane. The horizontal grey line denotes the LOQ of the UV-VIS measurement.

While the pore size reported for syringe filters indicates the average diameter of the filter pores, dialysis membranes are most commonly standardized by their MWCO. This parameter defines a molecular weight where 90% of a marker molecule was still retained by the membrane after 17 h [15]. For a 50 kDa dialysis membrane, we used BSA with a molecular weight of 66.5 kDa to measure the retention and quantified the protein by SEC-HPLC (see Section 2.3) and spectrophotometrically using a UV-Vis dip probe (Figure 4).

BSA was retained by the membrane for the first 8 h but steadily increased in concentration afterward (Figure 4). Commonly, the MWCO is determined at room temperature under mild agitation of the acceptor compartment only. Consequently, the stirring in the donor compartment, as well as the elevated temperature (37 °C), potentially accelerated the dialysis process. Our findings indicate that leakage from the donor compartment must be considered for the separation of molecules with a molecular volume close to the MWCO of the membrane. In a previous investigation, we reported the quantification of the drug-protein transfer as one potential application of the PTDR [13]. Importantly, these measurements can still be performed. Depending on the ratio between albumin and drug molecules, even a considerable leakage of BSA from the donor chamber does not lead to a corresponding error in the release profile. However, after 15 h and using a membrane with an MWCO of 50 kDa, the leakage would potentially affect the release. Therefore, such investigations should be carried out over a shorter period or using a smaller MWCO (e.g., 30 kDa) [13].

#### 3.2.3. Hydrodynamics in the Acceptor Compartment

**Figure 5:**
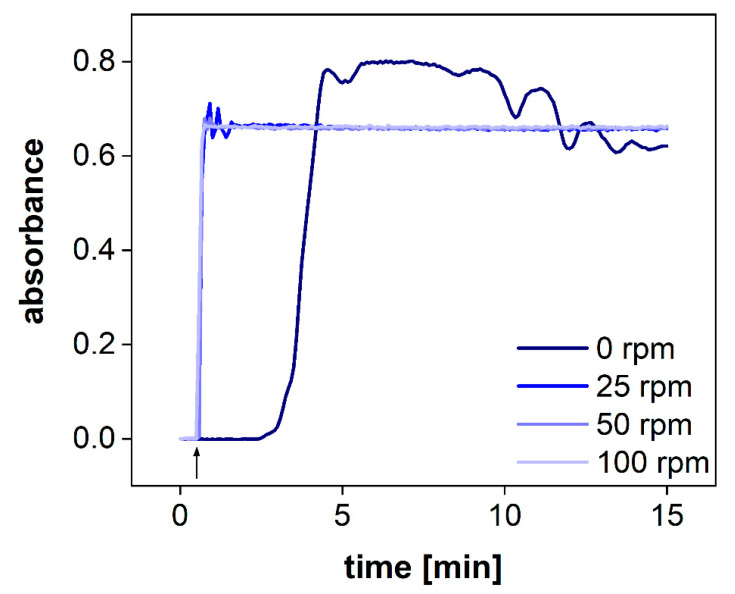
UV-Vis measurements of the acceptor compartment with an online dip probe at 283 nm at different stirring rates. The arrow denotes that the idebenone solution was spiked into the acceptor compartment.

The hydrodynamics in the acceptor compartment of the PTDR has a strong impact on the variability of the measured drug concentration. The common stirring rates of USP apparatus I/II are ranging from 25–100 rpm. To determine the impact of the stirring rate on drug distribution in the acceptor compartment, we utilized a dip probe and measured the absorbance of the release medium in a fixed position after the addition of a colored dye to the acceptor compartment. The dip probe enables measurement in real-time. Different stirring rates of 0, 25, 50, and 100 rpm were tested (Figure 5). Even at a stirring rate of 25 rpm, the absorbance remained constant after 1 min. Small fluctuations were observed during this first minute only. At 50 and 100 rpm, no fluctuations were observed. Without agitation, no steady absorbance was measured. After 15 min, the absorbance was close to the plateau observed for the other vessels.

Consequently, agitation of the acceptor compartment is required for the performance test. However, considering a common sampling time of 2.5–5 min, all stirring rates (25–100 rpm) enable accurate detection of the drug release.

#### 3.2.4. Permeation Rate at Various Stirring Rates

**Figure 6:**
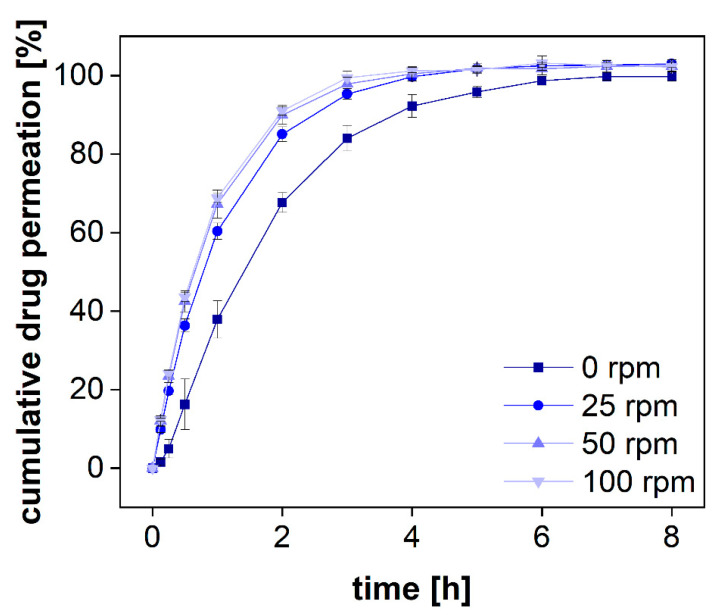
Mean permeation profiles of a diclofenac sodium solution at 37 °C using an RC dialysis membrane (MWCO 50 kDa). Stirring rates were changed accordingly (*n* = 6). Mean ± SD is presented.

The PTDR has the unique feature of accelerating dialysis processes by stirring the acceptor and the donor compartment. Floating, sedimentation, and agglomeration as well as the formation of layer structures in the donor chamber are common challenges of conventional dialysis experiments [9,16,17]. Although constant agitation does not always reflect the physiological environment of the administration site, it significantly reduces these effects and leads to improved reproducibility of the measurement [15,28]. Furthermore, the stirring accelerates the membrane transport and leads to a higher sensitivity when measuring kinetic processes. In dialysis, a sensitive measurement can only be achieved for dialysis rates exceeding the release rate. To measure this effect for various stirring rates, we dialyzed a solution of diclofenac sodium at 0, 25, 50, 100 rpm (Figure 6). This time-resolved permeation experiment is known as the release response test (RRT) and provides information on the separation time required by this method [29].

Without stirring, diclofenac permeated through the dialysis membrane very slowly and there was a lag time of several minutes before steady-state permeation was reached (Figure 6). Furthermore, the permeated amount was approximately 20% lower during the release phase and reached a plateau after 6 h. On the contrary, under constant stirring, the plateau was reached after 4 h only. This separation time represents the time until 100% of the total dose has been dialyzed. To estimate the sensitivity of the assay, the dialyzed fraction per time must be taken into consideration. For all stirring rates, a fraction corresponding to approximately 10% of the total dose was dialyzed within 7.5 min. The stirring rate of 25 rpm led to a slightly lower permeation rate as compared to 50 rpm. This was also reflected by a lowered membrane permeation rate constant. A further increase to 100 rpm did not affect the permeation rate significantly. However, these parameters are strongly affected by other parameters, such as the drug substance, the MWCO, and the membrane material (50 kDa, RC). Therefore, general recommendations are difficult to make. The stirring rate must be selected considering the membrane permeation and stability of each formulation and compound. Still, the present investigation indicates that stirring rates of more than 50 rpm may not generally improve the outcome for every membrane type. Furthermore, higher stirring rates are likely to increase the shear stress in the donor chamber and may lead to a change in the release mechanism. Therefore, depending on the sensitivity of the formulation to shear stress, common stirring rates between 25 and 50 rpm are preferred.

#### 3.2.5. Selectivity of the Assay for Specific Size Fractions

The BSA molecule binds diclofenac with high affinity through ionic and hydrophobic interactions using two unspecific binding sites [30]. A direct comparison between BSA and human serum albumin (HSA) resulted in minor differences between these two proteins, and the plasma protein binding in humans was reported to be 99% [31]. Therefore, in equilibrium, diclofenac is expected to be bound to proteins at a stoichiometric ratio of 2:1 [30,32]. We used different BSA concentrations to bind a certain fraction of the drug and investigated the specificity of the separation method for this protein-bound fraction. With a molecular weight of 66.5 kDa, BSA is reliably retained in the donor chamber during the first 8 h (see Section 3.2.2).

**Figure 7:**
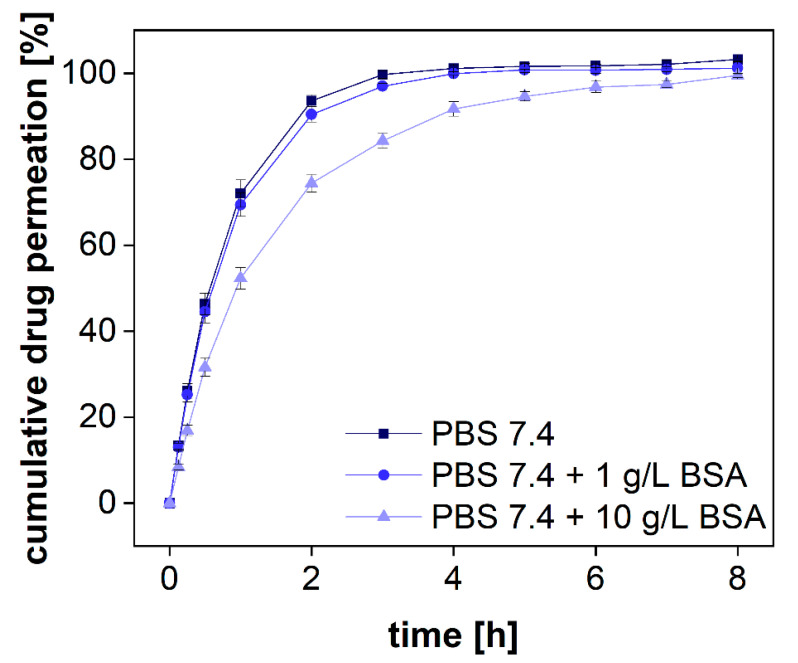
Cumulative permeation of diclofenac sodium with different amounts of BSA in PBS 7.4. A regenerated cellulose membrane (MWCO 50 kDa) was used to separate the two compartments. The experiment was conducted at 37 °C at 100 rpm (*n* = 6). Mean values ± SD are shown.

Following the standards of the European Pharmacopeia [33], two compendial buffer systems comprising 1 and 10 g/L of BSA were used, respectively. These concentrations are below the physiological albumin concentration of approximately 40 g/L. Diclofenac sodium was dissolved in PBS 7.4 comprising different amounts of protein. The permeation profiles were measured in the PTDR. The outcome of the investigation is presented in Figure 7. To quantitatively evaluate the difference in permeation, the area under the curve (AUC) was calculated for each permeation profile as well. As expected, at the lower BSA concentration, no significant difference between the profiles of protein-bound and free diclofenac was identified. At a concentration of 1 g/L, approximately 0.7% of diclofenac are bound to BSA whereas at a concentration of 10 g/L BSA approximately 7% are in a protein complex. Considering the analytical error indicated by the standard deviation, the difference in the AUCs between both permeation experiments (in the presence and absence of BSA) reflected the theoretical ratio of 0.93. In the experiment, a ratio of 0.91 ± 0.02 was found (Table 3). Hence, the drug was reliably retained by the membrane enabling sensitive detection of the protein-bound and the unbound fraction.

Considering the increase in BSA permeability over time, the duration of such experiments should not exceed 15 h. Afterward, an increasing analytical error is to be expected. Alternatively, smaller membrane pore sizes should be selected. For small molecules such as diclofenac, an MWCO of 20–30 kDa would still allow efficient separation of the two size fractions and may not be affected by the change in permeability to a similar extent.

### 3.3. In Vitro Performance Testing Using the PTDR

While the first set of experiments (Section 3.2) included procedures to assess the technical performance of the instrument, more studies were conducted to challenge the device using a common separation problem. Semisolid dosage forms often contain highly adhesive gelation agents and lipids likely to impact the dialysis process. Therefore, we selected two semisolid drug products, Voltaren^®^ Emulgel and Olfen^®^ gel, for this investigation and studied the influence of the excipients on membrane permeation. The data analysis was carried out using a model-dependent normalization procedure described previously (see Section 2.4) [5,12,13,14,15].

#### 3.3.1. Evaluation of the Influence of Excipients on Drug Permeation

**Figure 8:**
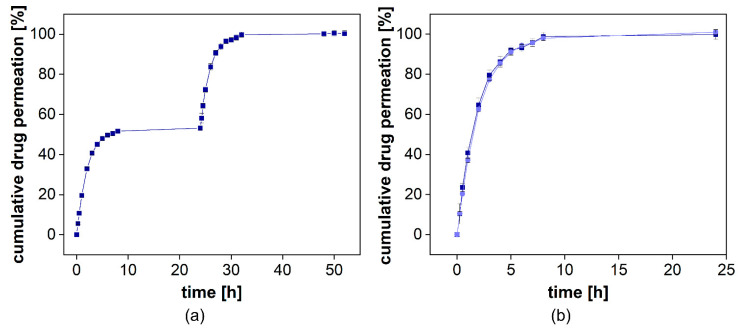
Cumulative release of diclofenac-DEA from Voltaren Emulgel^®^ with diclofenac-DEA solution spiked into the donor compartment after 24 h. Mean ± SD is presented (*n* = 3). On the left, the release profile over 48 h is presented (**a**). On the right, the overlay of the initial release and the permeation profile are provided (**b**).

To study the influence of excipients on drug permeation, the formulation Voltaren^®^ Emulgel was added to the donor chamber of the PTDR and a release experiment was performed (Figure 8a). Of the two semisolid formulations, the emulsion system was more likely to interact with the membrane. After 24 h, a solution of diclofenac was added to the donor chamber and dialyzed under similar conditions (Figure 8a). At neutral pH, the complete dissociation of the weak acid diclofenac increases the aqueous solubility of the drug substance dramatically. Therefore, during the initial 24 h, a rapid dissolution-driven release from the commercial formulation was observed.

When adding diclofenac in solution, there was no significant difference between the release and the permeation profiles observed (Figure 8b). This indicates that the formulation had no impact on the dialysis rate. While for most dialysis processes, a strong influence of semisolids on drug separation can be assumed, the PTDR effectively inhibits these membrane interactions and leads to a more reliable release test.

#### 3.3.2. Documentation of the Experimental Parameters

Many dialysis experiments reported in the literature do not provide accurate documentation of the exact experimental procedures. In the present investigation, we lay out a recommended methodology. Technical parameters to be reported are summarized in Table 4. They include common information such as the MWCO, or the membrane material, but also an exact description of the donor and the acceptor volume, as well as the preconditioning protocol used for this type of membrane.

#### 3.3.3. Data Analysis Using Model-Dependent Profile Correction

The membrane permeation rate constant (*k_m_*) is a performance indicator for the dialysis process and the separation of released drugs from the formulation. It represents an important analytical error and is therefore determined in a permeation experiment (Table 5). The four-step model uses this parameter to normalize the drug release profiles; however, for the application of dialysis-based release experiments in quality control, a specification range for *k_m_* would allow the preselection of dialysis membranes to improve reproducibility. Also, normalized release profiles can be directly compared even when measured with membranes from various vendors.

**Figure 9:**
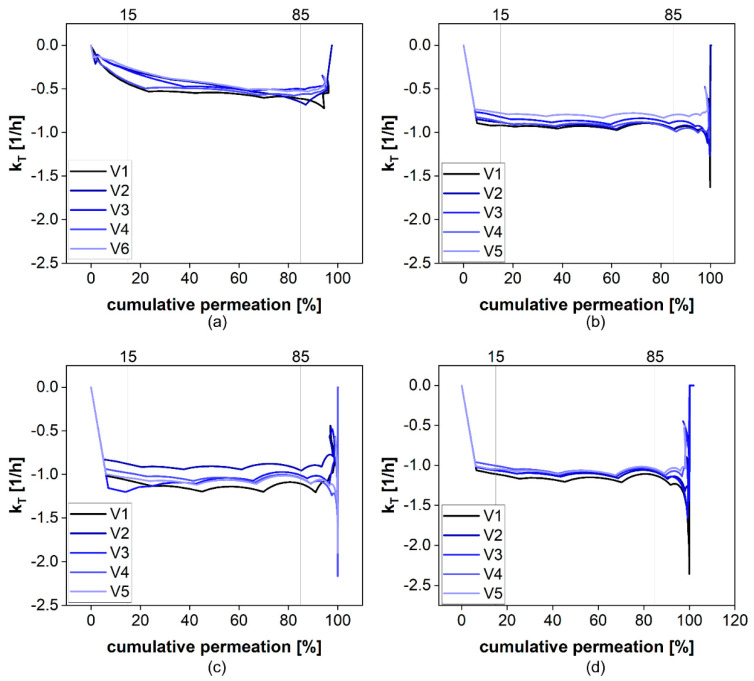
The calculated *k_T_* values at 0 (**a**), 25 (**b**), 50 (**c**), and 100 rpm (**d**).

For the calculation of *k_m_*, the total permeation rate constant *k_T_* is calculated for each time point of the permeation profile. Fluctuations in *k_T_* are more likely at the beginning and the end of the permeation experiment due to the initial distribution of the drug as well as the inaccuracies associated with the small concentration differences in the plateau phase. The *k_T_* values obtained from the permeation profiles of diclofenac at different stirring rates are presented in Figure 9. For the permeation range from 15 to 85% of the total dose, there were almost no fluctuations observed. Therefore, we decided to use this range for the calculation of *k_m_*. Evidently, the fluctuations are more pronounced for higher stirring rates, indicating a certain influence of the hydrodynamics on the diffusion rate.

To provide a convenient solution, we automated the process of *k_m_* calculation and published a user-friendly calculator (DIMEC) under the Creative Commons License. The operator provides the permeation profile, followed by an automated calculation of *k_m_*. The default settings include the technical parameters of the PTDR, however, other methodologies can be used. The user interface enables the customization of all the input parameters, including the surface area and the volume of the release cell.

#### 3.3.4. Validation of the Mathematical Model

**Figure 10:**
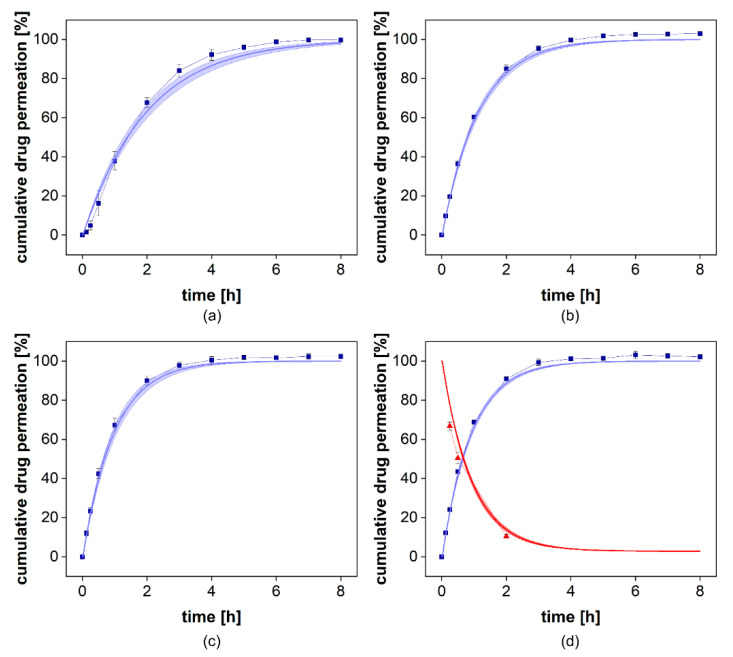
Comparison of observed and simulated permeation profiles at 0 (**a**), 25 (**b**), 50 (**c**), and 100 rpm (**d**). The blue squares indicate the mean ± SD (*n* = 6) observed cumulative drug permeation and the blue line presents the simulated cumulative drug permeation using the mean *k_m_* ± SD. In (**d**) the donor compartment was evaluated. The red triangles indicate the observed mean ± SD (*n* = 6) with the corresponding simulated relative donor concentration as the red line.

To provide evidence for the accuracy of the STELLA model, we compared the permeation profile with the simulated permeation profile represented by the *k_m_* value. It indicates the quality of the curve fit. The absolute average fold error (AAFE) is a simple measure of the difference between the simulated and the observed values in a simulation [11,14,24,25,26,27]. An AAFE of 1 indicates two identical profiles and values below 2 indicate a successful simulation [26,27]. For the permeation experiments carried out at stirring rates of 25–50 rpm, an AAFE of 1.03 was achieved (Table 5). At a stirring rate of 0 rpm, the lag time between injection of the solution and the constant drug flux through the membrane led to an increase in the AAFE to 1.27. At the highest stirring rate, the most considerable influence of the stirring rate on permeation was as expected. This still resulted in an accurate reflection of the permeation profile with an AAFE of 1.03. To validate our simulation, we collected samples from the donor compartment at the highest stirring rate after 0.25, 0.5, and 2 h and compared the observed and simulated drug concentrations. This is presented in Figure 10 (d, red line/red triangles). It provides further evidence for the reliability of the mathematical model.

The *k_m_* values at 0 and 25 rpm were 0.99 × 10^−3^ cm^2^/h and 1.76 × 10^−3^ cm^2^/h, respectively (Table 6). At 50 and 100 rpm, the permeation rates increased to 2.13 × 10^−3^ cm^2^/h and 2.17 × 10^−3^ cm^2^/h, respectively (Table 5). This confirms the contribution of the stirring rate to membrane transport that is responsible for the higher sensitivity of this release assay. However, at higher stirring rates, the influence was negligible. Therefore, stirring rates of more than 50 rpm should not be selected without confirming the benefit for a specific formulation or membrane pore size.

The average permeation rate constants for each stirring rate (Table 5) were calculated from 5 vessels and included 5–6 measured time points, depending on the number of sam-pling time points falling into the range of 15–85% permeation. For the statistical evalua-tion, we considered only time points sustained by quantification and not the extrapolated time points (>100). A significant difference in the membrane permeation rate constant was observed between all *k_m_* values except for the difference between 50 and 100 rpm (ANOVA, *p* > 0.05).

#### 3.3.5. Performance Testing Using Two Semisolid Diclofenac Formulations

The release of diclofenac is controlled by the aqueous solubility of each salt in the medium. Under sink conditions, there was no significant difference between the two formulations expected. For the performance assay, we selected a slightly acidic pH value corresponding to the pH of the human skin (5–6) [34]. Noteworthy, the conditions of the assay, including hydrodynamics, liquid volume, and buffer capacity, do not reflect the topical route of administration. Still, the release profiles provide information on the differences between these two dosage forms and could be used for excipient selection. The composition of the two gels is summarized in Table 6. Olfen^®^ gel comprises a conventional linear colloidal hydrogel structure with a certain amount of isopropyl alcohol embedded into the gel structure, whereas Voltaren Emulgel^®^ is a gelled oil-in-water emulsion. The drug was dissolved in the aqueous phase.

The cumulative drug release from both formulations is presented in Figure 8a. Voltaren^®^ Emulgel released diclofenac more rapidly compared to the Olfen^®^ gel and, within 48 h, reached a plateau at almost 60%, corresponding to the aqueous solubility determined for this release medium at 32 °C (0.0314 ± 0.0008 mg/mL for diclofenac sodium and 0.0332 ± 0.0020 mg/mL for diclofenac-DEA). Olfen^®^ gel released the drug much slower and reached a plateau at approximately 40%. The emulsifying agents present in Voltaren^®^ Emulgel as well as the improved solubility of diclofenac-DEA are the most likely explanation. A delaying effect of the emulsion system on dialysis could not be detected. Furthermore, it is unlikely that, when using the highly soluble salt of diclofenac, the emulsion system had a strong impact on the drug release.

Subsequently, we calculated the *k_m_* values using the DIMEC programmed in STELLA [19]. The permeation rate constants of the diclofenac sodium and diclofenac-DEA were 1.54 ± 0.12 × 10^−3^ cm^2^/h and 1.75 ± 0.19 × 10^−3^ cm^2^/h, respectively. Subsequently, we normalized the release profiles for each formulation using the PTDR ReNo. This calculator is provided under Creative Commons License as well.

**Figure 11:**
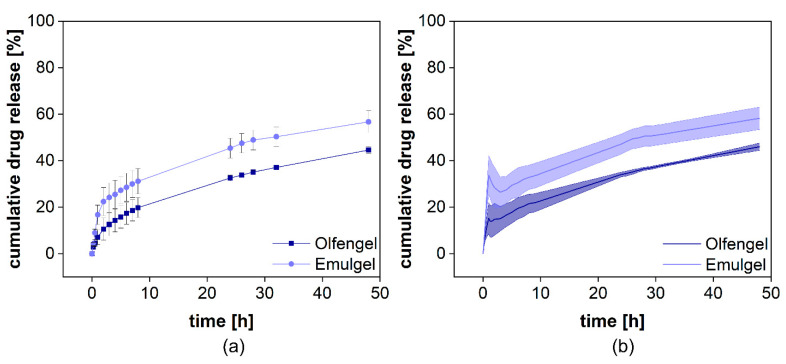
Cumulative release of two different diclofenac semisolid formulations. Measured diclofenac released into the acceptor compartment ((**a**), *n* = 4) and normalized release profile (**b**). Mean ± SD is shown.

After normalization (Figure 11b), a burst release of 16% from Olfen^®^ gel and 34% from Voltaren^®^ Emulgel were observed. It is evident that the release behavior of diclofenac from both hydrogels was widely driven by drug solubility and that the emulsion system represents a minor influence on the release.

## 4. Conclusions

Most dialysis-based performance assays demand a higher level of understanding from the operator. However, the requirements do not differ considerably from other dissolution tests. The selectivity of the separation method for the dissolved drug (e.g., by separating molecules with a specific molecular volume) and the separation time commonly have a strong impact on the release profile. Since most dialysis methods reported in previous research do not control hydrodynamics in the donor chamber and other influences, their limited reliability is not surprising. In the present investigation, we provide evidence for the reliability of a well-designed experimental setup, together with a dedicated mathematical methodology and documentation. To make it easier to comply with these high standards, we provide two computer models that can be customized for other dialysis-based release tests as well.

## Figures and Tables

**Table 1:** Parameters used for the DLS measurement.

Parameter	Description	Value
Refractive index	Gold nanoparticles	0.200
Absorbance	Gold nanoparticles	3.320
Dispersant temperature	Water	25.0 °C
Refractive index of the dispersant	Water	1.330
Viscosity of the dispersant	Water	0.8872
Measurement angle	Backscatter	173°
Positioning method	Fixed position	4.65
Repeated measurements	-	3

**Table 2:** Equilibrium solubility after 24 h of two diclofenac salts in different release media. Experiments were conducted in triplicate (*n* = 3). The values are expressed as mean ± SD.

Release Medium	pH	Temperature	Diclclofenac Sodium Salt [mg/mL]	Diclofenac-DEA Salt [mg/mL]
PBS	7.4	37.0 °C	14.2 ± 0.3	-
PBS + 1 g/L BSA	7.4	37.0 °C	15.2 ± 0.3	-
PBS + 10 g/L BSA	7.4	37.0 °C	18.5 ± 0.2	-
40 mM Acetate buffer	5.3	32.0 °C	0.0314 ± 0.0008	0.0332 ± 0.0020
10 mM Phosphate buffer	7.4	32.0 °C	13.5 ± 0.8	10.2 ± 0.2

**Table 3:** Calculated AUC of diclofenac sodium permeation in PBS 7.4 with different BSA amounts (*n* = 6).

Buffer	AUC (0–8 h) [µg × h/mL]	SD [µg × h/mL]	Ratio
PBS 7.4	292	2	-
PBS 7.4 + 1 g/L BSA	292	2	1.00 ± 0.01
PBS 7.4 + 10 g/L BSA	265	4	0.91 ± 0.02

**Table 4:** Important experimental parameters to be reported.

Parameter		Value
Membrane characteristics	Membrane material	Regenerated cellulose
	MWCO	50 kDa
	Membrane thickness	0.0065 cm
	Membrane tube flat width	28 mm
	Storage conditions	Prewetted in 0.5% sodium azide at 2–8 °C
	Preconditioning protocol of the	Rinse with water and soak for 30 min in water
	manufacturer	Repligen (USA)
Dispersion Releaser	Volume of donor compartment	3.4 mL
	Volume of acceptor fluid (Before and after the experiment)	120 mL (mini-vessel configuration)
	Surface area of the donor cell	10.95 cm^2^
Test conditions	Composition of the release medium	Various media tested (Materials and Methods)
	Temperature	32 °C or 37 °C (Materials and Methods)
	Stirring rate	0–100 rpm (Materials and Methods)
	Sampling volume	200 µL
	sampling time points	Various (Materials and Methods)
Sampling injection protocol	Reference experiment	A volume of 3.4 mL of an aqueous diclofenac solution
	Semisolid	Amount of each semisolid corresponding to 5 mg of diclofenac was weighed into the DR and the donor chamber was filled with release medium to a total volume of 3.4 mL.

**Table 5:** Permeation rates calculated for diclofenac sodium at different stirring rates. Each calculation is based on 5 vessels and included 5–6 time points (*n* = 25 or *n* = 30).

Stirring Rate	Permeation Rate Constant [cm^2^/h]	SD [cm^2^/h]	AAFE
0 rpm	0.99 × 10^−3^	0.9 × 10^−3^	1.27
25 rpm	1.76 × 10^−3^	0.10 × 10^−3^	1.03
50 rpm	2.13 × 10^−3^	0.18 × 10^−3^	1.03
100 rpm	2.17 × 10^−3^	0.09 × 10^−3^	1.03 ^1^ 1.23 ^2^

^1^ Evaluation of the acceptor compartment over the entire permeation profile. ^2^ Evaluation of the donor compartment at three time points (0.25, 0.5, 2 h).

**Table 6:** List of excipients of Voltaren^®^ Emulgel and Olfen^®^ gel.

Excipient	Voltaren^®^ Emulgel	Olfen^®^ Gel
Lactic acid		X
Diisopropyladipate		X
Isopropylalcohol	X	X
Sodium metabisulfite		X
Hydroxyethylcellulose		X
Hydroxypropylcellulose		X
Diethylamine	X	
Propylene glycol	X	
Mineral oil	X	
Cocoyl caprylocaprate	X	
Polyoxyl-20-cetostearylether	X	
Carbomer	X	
Perfume creme	X	
Purified water	X	X

## Data Availability

Not applicable.

## Supplementary Materials

The following are available online at https://www.mdpi.com/article/10.3390/pharmaceutics13122007/s1. Table S1: Commercial standards evaluated for testing the leakage of particles from the PTDR.

## Abbreviations

AAFE	absolute average fold error
AUC	area under the curve
BSA	bovine serum albumin
diclofenac-DEA	diclofenac diethylamine salt
DIMEC	Dialysis Membrane Permeation Calculator
DLS	dynamic light scattering
EV	extracellular vesicles
HPLC	High-performance liquid chromatography
HSA	human serum albumin
LOD	limit of detection
LOQ	limit of quantification
MWCO	molecular weight cut off
NIR	near-infrared light
PBS	phosphate-buffered saline
Ph. Eur.	European Pharmacopeia
PTDR	Pharma Test Dispersion Releaser
PTDR ReNo	PTDR release normalizer
PTFE	polytetrafluoroethylene
RC	regenerated cellulose
rpm	revolutions per minute
RRT	release response test
SD	standard deviation
SDS	sodium dodecyl sulfate
SEC	size exclusion chromatography
STELLA	Systems Thinking, Experimental Learning Laboratory with Animation
USP	United States Pharmacopeia
UV	ultraviolet light
Vis	visible light
