# Supplementary material for: An Update to Dialysis-Based Drug Release Testing—Data Analysis and Validation Using the Pharma Test Dispersion Releaser
Source: Pharmaceutics. 2021 Nov 25;13(12):2007. doi: 10.3390/pharmaceutics13122007 (PMC8708653; doi:10.3390/pharmaceutics13122007)
Supplement: Supplementary file 1 [file pharmaceutics-13-02007-s001.zip › pharmaceutics-1479723-supplementary.pdf]

pharmaceutics

Supplementary Materials: An Update to Dialysis-Based Drug Release Testing—Data Analysis and Validation Using the Pharma Test Dispersion Releaser

Marc-Phillip Mast, Harshvardhan Modh, Julian Knoll, Elena Fecioru and Matthias G. Wacker

Supplementary information

Table S1. Particle standards evaluated for testing the leakage of particles from the PTDR.

**Commercial standard**	**Related problem**	**Reference**
FluoroMax Green Fl.Markosph.<br>Particle diameter 0.05µm	Release (chemical stability)	Distrilab Particle Technology B.V. (Leusden, Netherlands)
FluoroMax Red Fl.Microsph.<br>Particle diameter 0.05µm	Release (chemical stability)	Distrilab Particle Technology B.V. (Leusden, Netherlands)
micromer®-redF<br>Particle diameter 0.1µm	Release (chemical stability)	Micromod Partikeltechnologie GmbH (Rostock, Germany)
FITC-CM-Dextran<br>Molecular weight 150,000	Release (chemical stability)	Sigma-Aldrich<br>(St. Louis, MO, USA)
**Non-commercial standard**	**Related problem**	**Reference**
Poly-lactide-*co*-glycolide loaded with Lumogen Yellow®<br>Particle size 0.2 µm	Release (dissociation)	10.1088/0957-4484/22/24/245102
